# Risk factors and a nomogram model for postoperative delirium in elderly gastric cancer patients after laparoscopic gastrectomy

**DOI:** 10.1186/s12957-022-02793-x

**Published:** 2022-09-29

**Authors:** Jie Chen, Xiaoli Ji, Hailin Xing

**Affiliations:** Department of Anesthesiology, Taizhou People’s Hospital Affiliated to Nanjing Medical University, No. 399 Hailing South Road, Taizhou City, 225300 Jiangsu Province China

**Keywords:** Gastric cancer, Elderly, Postoperative delirium, Nomogram model, Risk factors

## Abstract

**Background:**

To evaluate the risk factors of postoperative delirium (POD) in elderly gastric cancer (GC) patients after laparoscopic gastrectomy and construct a predictive model.

**Methods:**

Elderly GC patients undergoing laparoscopic gastrectomy were enrolled and grouped based on the status of POD development within postoperative 7 days. Independent risk factors were selected out by univariate and multivariate logistic regression analyses and then enrolled in the nomogram prediction model.

**Results:**

A total of 270 elderly GC patients were enrolled, and POD occurred in 74 (27.4%) patients within postoperative 7 days. The results of multivariate regression analysis indicated that age (*OR*: 3.30, 95% *CI*: 1.41–6.85, *P* < 0.001), sleeping pills (*OR*: 1.87, 95% *CI*: 1.12–3.09, *P* = 0.012), duration of ICU stay (*OR*: 1.55, 95% *CI*: 1.02–2.37, *P* = 0.029), albumin/fibrinogen ratio (AFR) (*OR*: 1.74, 95% *CI*: 1.03–2.76, *P* = 0.019), and neutrophils/lymphocytes ratio (NLR) (*OR*: 2.12, 95% *CI*: 1.11–4.01, *P* = 0.016) were five independent risk factors for POD in elderly GC patients. The AUC of the constructed nomogram model based on these five factors was 0.807.

**Conclusions:**

This study highlighted that age, AFR, NLR, sleeping pills taking, and duration of ICU stay were independent risk factors for POD, and the nomogram model based on these factors could effectively predict POD in elderly GC patients.

## Introduction

Gastric cancer (GC) is the fifth most common cancer worldwide with the highest incidence rates in Eastern Asia [1]. Especially in China, GC is the third most common cancer and becomes the second leading cause of cancer deaths [2, 3]. In addition, GC is often diagnosed at advanced stage in China with poor prognosis [4]. Surgical resection is the primary curative therapeutic strategy for GC. Postoperative complications after GC surgery are known to have serious effects on patient prognosis and quality of life [5, 6]. Postoperative delirium (POD) is a very common and serious complication, especially in elderly hospitalized patients [7]. POD usually occurs within postoperative 1–3 days, and its incidence can reach as high as 17–61% in elderly patients undergoing complicated or emergency surgeries [8, 9]. POD is well recognized as a serious complication and an independent predictor of worse prognosis [10]. POD is associated with increased medical costs, functional impairment, cognitive dysfunction, morbidity, and even mortality [11, 12]. Thus, it is important to determine risk factors of POD for prognosis improvement. Despite a considerable number of studies into POD, the reported risk factors for POD varied greatly in different studies. Thus, we aimed to investigate potential risk factors and to construct a potential individually nomogram prediction model for POD.

## Material and methods

### Patients

This is a single-center, retrospective study with the ethical approval of our hospital in accordance with the Declaration of Helsinki. Elderly GC patients undergoing laparoscopic gastrectomy between January 2018 and January 2022 were enrolled. Inclusion criteria are as follows: (1) age between 65 and 85 years, (2) with postoperative histopathologic diagnosis of GC, and (3) undergoing laparoscopic radical resection. Exclusion criteria are as follows: (1) undergoing laparotomy or conversion to laparotomy, (2) with preoperative delirium or other cognitive impairment, (3) with preoperative adjuvant therapy (e.g., chemotherapy), (4) with incomplete data, and (5) refused or unable to cooperate.

### Data collection

The data were collected as follows: (1) demographics, including age, body mass index (BMI), gender, American Society of Anesthesiologists (ASA) grade, education level, and current smoking and drinking habits; (2) clinical variables, including history of abdominal surgery, preoperative medications, preoperative anxiety, surgical APGAR score, and ECOG status; (3) surgical pathology data, including types of surgery, operation time, recovery time, estimated blood loss, tumor location, lymph node dissection, pathological TNM stage, and duration of ICU stay; (4) preoperative laboratory tests, including hemoglobin (Hb), white blood cell (WBC), platelet (Plt), urea, creatinine (Cr), albumin (Alb), fibrinogen (Fib), neutrophils (N), and lymphocytes (L); and (5) tumor biomarkers, including carcinoma embryonic antigen (CEA), CA19-9, CA72-4, and CA125.

### Outcomes and definitions

Albumin/fibrinogen ratio (AFR) was calculated with Alb divided by Fib, while neutrophils/lymphocytes ratio (NLR) with N is divided by L. Based on the Chinese version of Zung’s Self-Rating Anxiety Scale (SAS), patients with a SAS score ≥ 50 were defined as anxiety [13]. The primary outcome is the incidence of POD within postoperative 7 days. The diagnosis of POD was made according to the criteria of the 5th edition of *Diagnostic and Statistical Manual of Mental Disorders* (DSM-5, 2013) [14]. As described previously, POD was diagnosed using a retrospective chart review method [15, 16]. All the medical and nursing records within postoperative 7 days were systematically checked by two independent anesthetists, to identify the presence of DSM-V criteria for POD. As reported previously [17], the surgical Apgar score was calculated by intraoperative estimated blood loss, the lowest heart rate, and mean arterial.

### Statistical analysis

Statistical analyses were performed with GraphPad Prism v8.0 (GraphPad Inc., CA, USA) and SPSS v23.0 (SPSS Inc.). Data are presented as number with percentage (*n*, %) or mean ± standard deviation (SD). Data analyses between groups were performed with the methods of Student *t*-, Mann-Whitney *U*-, or chi-square tests. Binary univariate and multivariate logistic regression analyses were performed to evaluate potential risk factors associated with POD. The predictive values of continuous variables were evaluated using the receiver operating characteristic (ROC) curve. R v4.0 was used to construct and evaluate the nomogram prediction model. A two-sided *P* < 0.05 was considered statistically significant.

## Results

According to the inclusion and exclusion criteria, a total of 270 elderly GC patients were enrolled in the data analysis. The mean age of the entire cohort was 73.4 years, and the majority (65.9%, 178/270) were male patients. Within postoperative 7 days, POD occurred in 74 (27.4%) of the 270 patients. The detailed demographics and clinical information of patients are available in Table 1. The mean age (*P* < 0.001), ASA grade (*P* = 0.023), and duration of hospital stay (*P* = 0.004) in the POD group were much higher than in the non-POD group. The proportions of patients with current drinking habits (*P* = 0.049), sleeping pills taking (*P* = 0.009), and preoperative anxiety (*P* = 0.021) were statistically higher in patients with POD than those without POD. In addition, patients with a longer duration of operation (*P* = 0.011), recovery (*P* = 0.039), and ICU stay (*P* = 0.002) were more likely to develop POD. No statistical differences were observed between POD and non-POD groups with respect to other demographic and clinical variables (*P* > 0.05).

**Table 1:** Demographic and clinical characteristics associated with POD in elderly GC patients

	POD		
Variables	No (*n* = 196)	Yes (*n* = 74)	*p*-value
Age (year)	72.5 ± 3.8	75.8 ± 3.8	< 0.001*
BMI (kg/m^2^)	20.8 ± 2.3	21.0 ± 2.5	0.534
Gender, *n* (%)	-	-	0.524
Male	127 (64.8)	51 (68.9)	-
Female	69 (35.2)	23 (31.1)	-
ASA physical status, *n* (%)	-	-	0.023*
I-II	158 (80.6)	50 (67.6)	-
III-IV	38 (19.4)	24 (32.4)	-
Smoking, *n* (%)	37 (18.9)	15 (20.3)	0.796
Drinking, *n* (%)	30 (15.3)	19 (25.7)	0.049*
Education level, *n* (%)	-	-	0.218
≥ High school	33 (16.8)	8 (10.8)	-
< High school	163 (83.2)	66 (89.2)	-
History of abdominal surgery, *n* (%)	47 (24.0)	21 (28.4)	0.458
Preoperative medications, *n* (%)	-	-	-
Antidiabetics	26 (13.3)	10 (13.5)	0.957
Antihypertensive drugs	38 (19.4)	12 (16.2)	0.550
Sleeping pills	19 (9.7)	16 (21.6)	0.009*
Preoperative anxiety, *n* (%)	25 (12.8)	18 (24.3)	0.021*
ECOG status, *n* (%)	-	-	0.094
0	140 (71.4)	45 (60.8)	-
≥ 1	56 (28.6)	29 (39.2)	-
Types of surgery, *n* (%)	-	-	0.560
Total gastrectomy	59 (30.1)	25 (33.8)	-
Partial gastrectomy	137 (69.9)	49 (66.2)	-
Operation time (h)	2.8 ± 0.6	3.0 ± 0.5	0.011*
Time to awakening (min)	38.2 ± 6.5	40.1 ± 7.3	0.039*
Estimated blood loss (ml)	150 (85)	160 (90)	0.172
Surgical APGAR score	6.2 ± 1.4	5.9 ± 1.5	0.125
Tumor location, *n* (%)	-	-	0.644
Upper 1/3	23 (11.7)	9 (12.2)	-
Middle 1/3	70 (35.7)	22 (29.7)	-
Low 1/3	103 (52.6)	43 (58.1)	-
Lymph node dissection	-	-	0.697
D0-D1	119 (60.7)	43 (58.1)	-
≥ D2	77 (39.3)	31 (41.9)	-
Pathological TNM stage	-	-	0.517
II	116 (59.2)	47 (63.5)	-
III	80 (40.8)	27 (36.5)	-
Duration of ICU stay (d)	1.8 ± 0.9	2.2 ± 1.0	0.002*
Duration of hospital stay (d)	11.8 ± 2.2	12.7 ± 2.4	0.004*

*POD* postoperative delirium, *GC* gastric cancer, *BMI* body mass index, *ASA* American Society of Anesthesiologists, *ECOG* Eastern Cooperative Oncology Group, *ICU* intensive care unit

*P-value < 0.05 by chi-square test, Fisher exact test, *t*-test, or Mann-Whitney *U*-test

The preoperative laboratory indexes are displayed in Table 2. Patients in POD group had a significant higher NLR (4.5 ± 2.0 vs 3.5 ± 1.3, *P* < 0.001) and lower AFR (9.7 ± 1.7 vs 10.4 ± 1.9, *P* = 0.006) than those in non-POD group. There were no statistical differences between patients with or without POD with regard to Hb, WBC, platelet, Cr, urea, CEA, CA19-9, CA72-4, and CA125 (*P* > 0.05).

**Table 2:** Preoperative laboratory tests associated with POD in elderly GC patients

	POD		
Patient characteristics	No (*n* = 196)	Yes (*n* = 74)	*p*-value
Hb (mg/dL)	12.0 ± 1.7	11.9 ± 1.8	0.672
WBC (× 10^9^/L)	7.5 ± 2.0	7.7 ± 1.8	0.452
Platelet (× 10^9^/L)	193 (82)	182 (80)	0.260
Cr (mg/dL)	0.91 ± 0.12	0.89 ± 0.11	0.213
Urea (mmol/L)	6.3 ± 1.0	6.4 ± 1.1	0.478
AFR	10.4 ± 1.9	9.7 ± 1.7	0.006*
NLR	3.5 (1.9)	4.7 (2.8)	< 0.001*
CEA (ng/ml)	-	-	0.440
≥ 5.0	20 (10.2)	10 (13.5)	-
< 5.0	176 (89.8)	64 (86.5)	-
CA19-9 (kU/L)	-	-	0.228
≥ 40	19 (9.7)	11 (14.9)	-
< 40	177 (90.3)	63 (85.1)	-
CA72-4 (U/mL)	-	-	0.455
≥ 6	25 (12.8)	7 (9.5)	-
< 6	171 (87.2)	67 (90.5)	-
CA125 (U/ml)	-	-	0.205
≥ 35	27 (13.8)	6 (8.1)	-
< 35	169 (86.2)	68 (91.9)	-

*POD* postoperative delirium, *GC* gastric cancer, *Hb* hemoglobin, *WBC* white blood cell, *Cr* creatinine, *AFR* albumin/fibrinogen ratio, *NLR* neutrophils/lymphocytes ratio, *CEA* carcinoembryonic antigen, *CA* cancer antigen

*P-value < 0.05 by chi-square test, Fisher exact test, *t*-test, or Mann-Whitney *U*-test

**Fig. 1:**
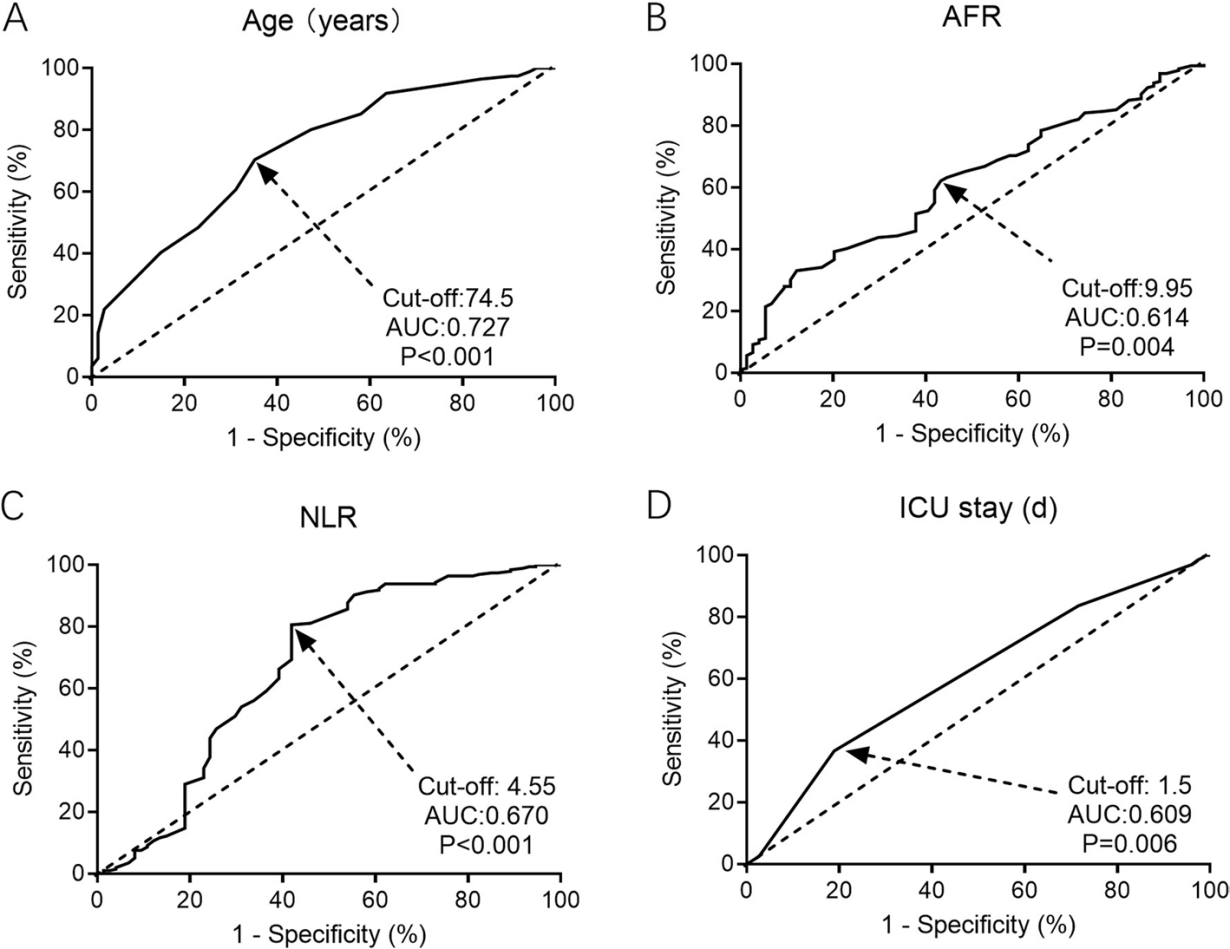
Predictors of POD by ROC curve analyses. **A** Age. **B** AFR. **C** NLR. **D** Duration of ICU stay. POD, postoperative delirium; ROC, receiver operating characteristic; AFR, albumin/fibrinogen ratio; NLR, neutrophils/lymphocytes ratio; ICU, intensive care unit; AUC, area under the curve

Subsequently, ten potential risk factors (*P* < 0.05 in Tables 1 and 2) were included in the univariate and multivariate logistic regression models. As shown in Table 3, age (*OR*: 3.30, 95% *CI*: 1.41–6.85, *P* < 0.001), sleeping pills (*OR*: 1.87, 95% *CI*: 1.12–3.09, *P* = 0.012), duration of ICU stay (*OR*: 1.55, 95% *CI*: 1.02–2.37, *P* = 0.029), AFR (*OR*: 1.74, 95% *CI*: 1.03–2.76, *P* = 0.019), and NLR (*OR*: 2.12, 95% *CI*: 1.11–4.01, *P* = 0.016) were five independent risk factors for POD in elderly GC patients. As revealed by the results of ROC curve analyses (Fig. 1), age (cutoff value: 74.5, *AUC*: 0.727, *P* < 0.001), duration of ICU stay (cutoff value: 1.5, *AUC*: 0.609, *P* = 0.006), AFR (cutoff value: 9.95, *AUC*: 0.614, *P* = 0.004), and NLR (cutoff value: 4.55, *AUC*: 0.670, *P* < 0.001) were four effective predictors of POD.

**Table 3:** Univariate and multivariate logistic regression analyses of POD

	Univariate		Multivariate	
Variables	OR (95% CI)	*p*-value	OR (95% *CI*)	*p*-value
Age	2.64 (1.16–5.67)	< 0.001*	3.30 (1.41–6.85)	< 0.001*
ASA physical status (III/IV vs I/II)	1.45 (0.96–2.19)	0.074	-	-
Drinking (yes vs no)	1.16 (0.54–2.44)	0.701	-	-
Sleeping pills (yes vs no)	1.81 (1.17–2.73)	0.009*	1.87 (1.12–3.09)	0.012*
Preoperative anxiety (yes vs no)	1.59 (1.04–2.45)	0.033*	1.72 (0.74–3.94)	0.210
Operation time	1.65 (1.07–2.54)	0.037*	1.57 (0.61–4.02)	0.344
Recovery time	1.27 (0.47–3.34)	0.587	-	-
Duration of ICU stay	1.81 (1.13–2.91)	0.015*	1.55 (1.02–2.37)	0.029*
AFR	1.77 (1.08–2.87)	0.013*	1.74 (1.03–2.76)	0.019*
NLR	2.21 (1.22–3.98)	0.008*	2.12 (1.11–4.01)	0.016*

*POD* postoperative delirium, *ASA* American Society of Anesthesiologists, *ICU* intensive care unit, *AFR* albumin/fibrinogen ratio, *NLR* neutrophils/lymphocytes ratio, *OR* odds ratio, *CI* confidence interval. *P-value < 0.05

**Fig. 2:**
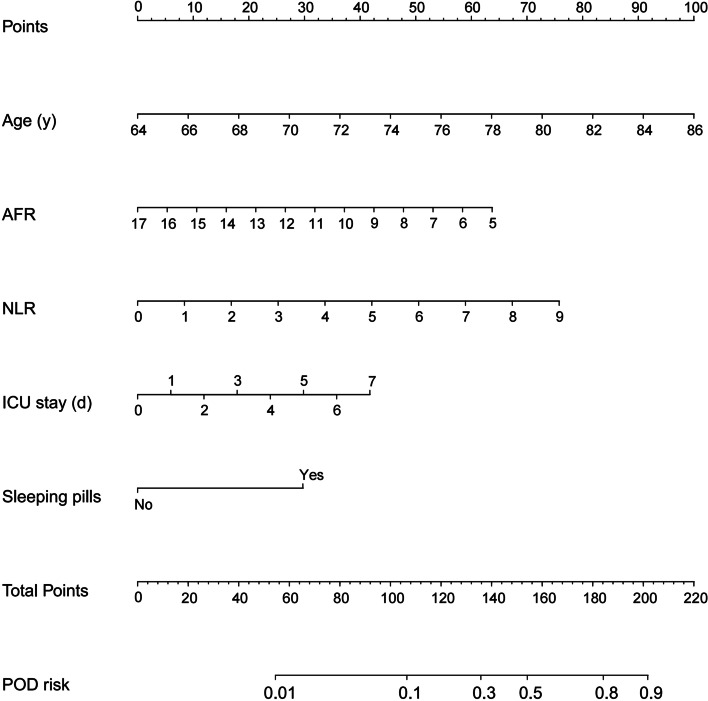
The nomogram prediction model for POD. POD, postoperative delirium; AFR, albumin/fibrinogen ratio; NLR, neutrophils/lymphocytes ratio; ICU, intensive care unit

**Fig. 3:**
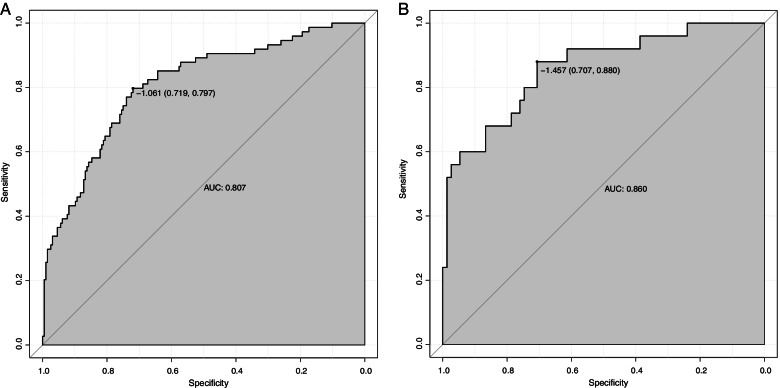
The evaluation of nomogram model for POD by ROC curve analysis in training (**A**) and validation (**B**) sets. POD, postoperative delirium; ROC, receiver operating characteristic; AUC, area under the curve

**Fig. 4:**
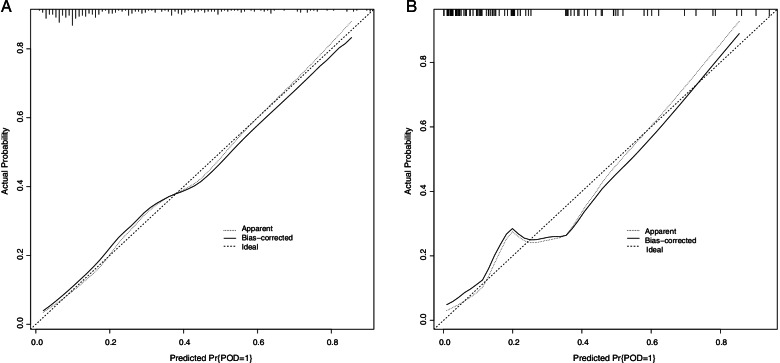
The evaluation of nomogram model for POD by calibration curve analysis in training (**A**) and validation (**B**) sets. POD, postoperative delirium

**Fig. 5:**
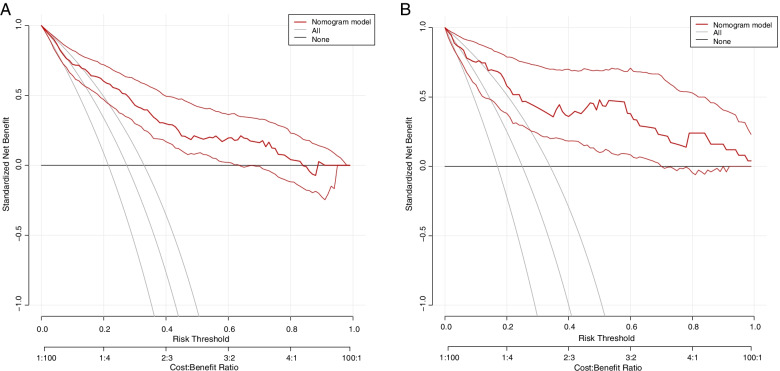
The evaluation of nomogram model for POD by DCA curve analysis in training (**A**) and validation (**B**) sets. POD, postoperative delirium; DCA, decision curve analysis

Based on the results of multivariate analysis, we constructed a nomogram prediction model with these five factors. As shown in Fig. 2, a nomogram prediction model based on these five factors was constructed to make more accurately personalized predictions for POD. The model was then validated both internally (training set, *n* = 270) and externally (validation set, *n* = 100) by R. The performed ROC curve analyses showed an AUC of 0.807 in training set (Fig. 3A) and 0.860 in validation set (Fig. 3B), indicating the well discriminative ability of this nomogram model. In addition, the calibration curve showed that this model did well compared with an ideal prediction model in both training (Fig. 4A) and validation (Fig. 4B) sets. Moreover, DCA curve was performed to evaluate the ability of the nomogram to improve clinical decision-making. DCA also demonstrated the clinical benefits of this nomogram model in both training (Fig. 5A) and validation (Fig. 5B) sets.

In addition, we investigated the correlation between other complications and POD. As shown in Table 4, the incidences of intestinal obstruction, gastroparesis, wound infection, bleeding, anastomotic leakage, pulmonary complications, and venous thrombosis were not statistically different between patients with or without POD (*P* > 0.05).

**Table 4:** Other postoperative complications associated with POD in elderly GC patients

	POD		
Complications	No (*n* = 196)	Yes (*n* = 74)	*p*-value
Intestinal obstruction, *n* (%)	8 (4.1)	2 (2.7)	0.732
Gastroparesis, *n* (%)	18 (9.2)	7 (9.4)	1.000
Wound infection, *n* (%)	13 (6.6)	3 (4.1)	0.569
Bleeding, *n* (%)	7 (3.6)	2 (2.7)	1.000
Anastomotic leakage, *n* (%)	5 (2.6)	2 (2.7)	1.000
Pulmonary complications, *n* (%)	10 (5.1)	5 (6.8)	0.563
Venous thrombosis, *n* (%)	7 (3.6)	2 (2.7)	1.000

*POD* postoperative delirium, *GC* gastric cancer

## Discussion

The incidence of POD of the entire cohort in this study is 27.4%, which was quite similar to the 26.1% by Choi et al. [18], higher than the 17.0% by Chen et al. [19], and 20.6% by Kinoshita et al. [20]. In addition, the incidence of POD in GC patients reported by Honda and his group [21] is as low as 4.5%. In our opinion, the different delirium diagnosis criteria, patient characteristics (especially age range), preoperative comorbidities, surgery types, and perioperative managements correspond to the different incidences among studies.

This study highlighted five independent risk factors (age, AFR, NLR, sleeping pills taking, and duration of ICU stay) for POD in elderly GC patients. An older age has been widely accepted as an independent risk factor for POD development in various studies [22–24]. Older patients have a greater probability of comorbidities, multiple medications taking, and cognitive impairment [22], which results in a significantly increased risk of POD. In addition, increasing age is also accompanied with the prevalence of frailty, which is more susceptible to POD [25]. A recent study by Jiang et al. [26] indicates AFR as an independent risk factor for POD in elderly patients after total joint arthroplasty. In addition, a recent retrospective study suggests that NLR is an independent predictor of poststroke delirium among patients with acute ischemic stroke [16]. AFR is a novel indicator reflecting inflammation and nutrition status [27], while NLR is reliably reflecting inflammation [28]. AFR and NLR were both widely used as prognostic indicators in various diseases [29, 30]. These studies strongly suggest a close association between inflammation and POD. The pathophysiology of delirium has not been fully elucidated until now, but the inflammation is believed to be at least partially involved in the mechanisms [31]. Moreover, the habitual use of sleeping pills (especially benzodiazepines) is reported as a risk factor for POD [32], which supports our conclusions. Additionally, a previous study indicates that prolonged ICU hospitalization is positively associated with delirium among ICU patients [33]. All these studies are quite in accordance with our results.

In order to prevent POD, it is critical to investigate potential preoperative risk factors. Based on the results of multivariate logistic analyses, this study constructed a nomogram prediction model. The results of model evaluation through ROC, DCA, and calibration curve analyses indicated that this nomogram model has a well predictive value with an AUC of 0.807. Therefore, this combined nomogram model may assist in individually POD risk evaluation, clinical decision-making, POD prevention, and outcome improvement.

This study has some limitations. First, it has inherent flaws of a retrospective single-center study. Second, our results need to be externally validated by further multicenter studies. Third, the nomogram model may be improved by enrolling some more important factors. Last, no clear consensus has been reached in the definition of POD, and this study only used the DSM V criteria.

## Conclusions

In conclusion, this study highlighted that age, AFR, NLR, sleeping pills taking, and duration of ICU stay were independent risk factors for POD, and the nomogram model based on these factors could effectively predict POD in elderly GC patients.

## Data Availability

Please contact the corresponding author for data requests.

## Abbreviations

GC: Gastric cancer
POD: Postoperative delirium
BMI: Body mass index
ASA: American Society of Anesthesiologists
ECOG: Eastern Cooperative Oncology Group
Hb: Hemoglobin
WBC: White blood cell
Plt: Platelet
Cr: Creatinine
Alb: Albumin
Fib: Fibrinogen
N: Neutrophils
L: Lymphocytes
CEA: Carcinoma embryonic antigen
AFR: Albumin/fibrinogen ratio
NLR: Neutrophils/lymphocytes ratio
SAS: Self-Rating Anxiety Scale
DSM: *Diagnostic and Statistical Manual of Mental Disorders*
SD: Standard deviation
ROC: Receiver operating characteristic
AUC: Area under the curve
OR: Odds ratio
CI: Confidence interval
